# Functional Characterization of AoFlbB and AoFlbD Reveals Their Roles in Sporulation and Trap Formation in *Arthrobotrys oligospora*

**DOI:** 10.3390/microorganisms14081676

**Published:** 2026-07-30

**Authors:** Yi Chen, Yanmei Shen, Hui Yuan, Qiyan Hu, Lihua Wei, Yuehui Li, Meichen Zhu, Jinkui Yang

**Affiliations:** Key Laboratory for Conservation and Utilization of Bio-Resources in Yunnan, Yunnan Key Laboratory of Basic Research and Innovative Application for Green Biological Production, School of Life Sciences, Yunnan University, Kunming 650091, China; 12024214011@stu.ynu.edu.cn (Y.C.); sym121350@163.com (Y.S.); yuanhui201228@163.com (H.Y.); qyhu1208@gmail.com (Q.H.); lihuaq202503@163.com (L.W.); 15839905978@163.com (Y.L.)

**Keywords:** nematophagous fungi, developmental transcription factors, asexual development, fungal morphogenesis, nematode predation efficiency

## Abstract

Sporulation is essential for asexual reproduction and dispersal in filamentous fungi, but the roles of upstream sporulation regulators in nematode-trapping (NT) fungi remain poorly understood. In this study, we characterized two Flb homologs, AoFlbB and AoFlbD, in *Arthrobotrys oligospora*. AoFlbB contains a bZIP-type domain, whereas AoFlbD contains a Myb-like DNA-binding domain, and both proteins are conserved among NT fungi. Deleting *AoflbB* or *AoflbD* had little effect on hyphal growth but markedly reduced spore production and altered spore germination, and real-time quantitative PCR analysis showed that the central sporulation regulators were significantly differently expressed, collectively indicating that AoFlbB and AoFlbD play positive roles in sporulation. Furthermore, both mutants produced more traps and showed enhanced nematode predation efficiency. The mutants also showed altered cell wall stress responses, hyphal cell length, nuclear number, endocytic trafficking, and metabolite profiles. The Δ*AoflbB* mutant showed irregular cell wall wrinkling, whereas the Δ*AoflbD* mutant displayed increased vesicle-like structures and accelerated FM4-64 uptake. Extracellular protease activity was comparable to the WT in Δ*AoflbB* but reduced in Δ*AoflbD*. Transcriptomic analysis of Δ*AoflbD* showed that the differentially expressed genes were significantly enriched in metabolism-related pathways. These findings indicate that AoFlbB and AoFlbD promote sporulation and contribute to the coordination of asexual reproduction and trap formation in *A. oligospora*.

## 1. Introduction

Plant-parasitic nematodes are among the most damaging agricultural pests, parasitizing varied staple and cash crops and causing substantial yield losses. Nematode-trapping (NT) fungi can produce specialized predatory structures, including constricting rings, adhesive networks, adhesive columns, and adhesive knobs, to capture nematodes, and they, therefore, represent promising biological resources for the sustainable management of plant-parasitic nematodes [1,2]. *Arthrobotrys oligospora* Fresen., as a representative NT fungus, is a model species for studying fungus–nematode interactions and lifestyle switching [3,4,5]. Under nutrient-limited or nematode-induced conditions, *A. oligospora* shifts from saprophytic growth to a predatory lifestyle, forming adhesive three-dimensional networks (traps) to immobilize, penetrate, and digest nematodes as nutrient sources [6,7].

Sporulation is central to asexual reproduction, dispersal, and environmental adaptation in filamentous fungi, and it is closely linked to infection-related development in many fungal pathogens [8,9,10,11]. In *Aspergillus nidulans* (Eidam) G. Winter, sporulation proceeds through vegetative growth, aerial hyphal formation, conidiophore differentiation, phialide formation, and spore maturation [8]. In plant-pathogenic fungi, spores often serve as infectious propagules that mediate dissemination and host invasion. For example, spores of the rice blast fungus *Magnaporthe oryzae* B.C. Couch germinate and form appressoria before invading plant tissues [9], whereas spores of *Fusarium* species are major agents of disease transmission [10]. In the opportunistic human pathogen *Aspergillus fumigatus* Fresen., airborne spores can be inhaled and cause severe disease in susceptible hosts [11]. Thus, defining the regulatory mechanisms that govern sporulation is important for understanding fungal development, dispersal, and infection-related processes.

*A. nidulans* is a well-established model for studying the regulation of asexual development in filamentous fungi. Its sporulation program is controlled by upstream developmental activators, the central BrlA–AbaA–WetA regulatory cascade, and environmental signals [8,12]. Upstream regulators, including FluG and FlbA–E, initiate asexual development largely through regulating *brlA* expression. The BrlA–AbaA–WetA cascade subsequently controls conidiophore differentiation, phialide function, spore maturation, and spore wall formation [13]. Environmental signals such as light also modulate asexual development in *A. nidulans*. Light-responsive proteins such as LreA, LreB, and CryA contribute to sporulation by influencing upstream developmental regulators and *brlA* expression [14].

FlbB and FlbD are key transcription factors in the upstream sporulation network of *A. nidulans*. FlbB is a bZIP-type transcription factor that accumulates at hyphal tips and translocates to nuclei during asexual development, and the deletion of *flbB* causes a typical fluffy phenotype and severe sporulation defects, indicating that FlbB is required for the initiation of asexual development [15]. *flbD* encodes a Myb-like DNA-binding protein involved in conidiophore initiation; the loss of *flbD* markedly impairs sporulation, whereas its overexpression promotes the onset of sporulation [16]. Further studies have shown that FlbB promotes *flbD* expression and cooperates with FlbD to activate *brlA* transcription, thereby linking upstream developmental signals to the central BrlA–AbaA–WetA cascade [17]. These findings establish FlbB and FlbD as important upstream regulators of asexual development in filamentous fungi.

As in many filamentous fungi, sporulation is a major means of asexual reproduction and environmental dispersal in *A*. *oligospora* [18]. Recent research has begun to define developmental regulators in *A*. *oligospora* that are homologous to components of the classical sporulation network in *A. nidulans*. The central regulators AoBrlA, AoAbaA, and AoWetA control sporulation and influence trap formation, pathogenicity, and secondary metabolism [19]. Upstream regulators have likewise been linked to these processes. AoSfgA physically interacts with AoFluG and positively regulates *AofluG* expression, whereas *AosfgA* or *AofluG* deletion affects hyphal development, sporulation, stress responses, vacuole assembly, and secondary metabolism [20]. In addition, several signaling and trafficking-related regulators, such as AoSsk1, AoRvs167, arrestin-family proteins, and AoSec22, have been reported to affect trap formation, pathogenicity, stress responses, endocytosis, vesicle trafficking, and secondary metabolism in *A*. *oligospora* [21,22,23,24]. These findings suggest that the sporulation regulatory network in *A. oligospora* is functionally connected with trap development and predatory lifestyle switching.

Besides transcriptional regulation, metabolic regulation is also closely associated with trap formation in *A. oligospora*. Previous genomic and proteomic analyses suggested that trap formation is accompanied by changes in amino acid metabolism, carbohydrate metabolism, cell wall and membrane biogenesis, and other cellular processes [4]. In addition, metabolomic studies have linked secondary metabolism and volatile signaling to trap morphogenesis and nematode attraction in *A. oligospora* [25,26]. These findings indicate that the predatory process is not only a morphological process but also involves coordinated cellular and metabolic remodeling.

Although Flb-type upstream developmental regulators including FlbB and FlbD have been well characterized in model filamentous fungi such as *A. nidulans*, their roles in NT fungi remain unclear. This gap is particularly relevant because sporulation and predatory development coexist in *A. oligospora*, where developmental regulators may coordinate asexual reproduction with trap formation and pathogenicity. In this study, we combined gene deletion, phenotypic analysis, metabolomic profiling, and transcriptomic analysis to characterize the roles of AoFlbB and AoFlbD in sporulation and trap formation in *A. oligospora*.

## 2. Materials and Methods

### 2.1. Strains, Plasmids, and Culture Conditions

*A. oligospora* ATCC 24927 was used as the wild-type (WT) strain. The Δ*AoflbB* (*AOL_s00078g317*) and Δ*AoflbD* (*AOL_s00097g46*) mutants were generated from this parental strain. All *A. oligospora* strains were routinely maintained on potato dextrose agar (PDA) at 28 °C. The plasmids pCSN44 and pRS426 were used to construct gene replacement vectors. pCSN44 served as the template for amplifying the hygromycin B resistance cassette (*hph*), and pRS426 was used as the yeast recombination vector. Both plasmids were propagated in *Escherichia coli* DH5α. The gene replacement fragment was assembled in *Saccharomyces cerevisiae* FY834 through homologous recombination, and yeast transformants were selected on SC-Ura medium and then propagated in yeast extract–peptone–dextrose (YPD) medium. *Caenorhabditis elegans* was cultured on sterile oat medium at room temperature and used for trap induction and nematode predation assays [21,24].

### 2.2. Bioinformatic Analysis of AoFlbB and AoFlbD

AoFlbB and AoFlbD homologs were identified via the NCBI BLASTP searches (https://blast.ncbi.nlm.nih.gov/, accessed on 20 October 2025) against the *A. oligospora* ATCC 24927 proteome [4] using reported FlbB and FlbD protein sequences from representative filamentous fungi as queries. BLASTP searches were performed with an E-value cutoff of 1 × 10^−5^, and candidate homologs were selected based on sequence similarity and conserved domain information. Two candidate proteins encoded by *AOL_s00078g317* and *AOL_s00097g46* were identified and designated as AoFlbB and AoFlbD, respectively. The physicochemical properties of AoFlbB and AoFlbD, including their theoretical molecular weights and isoelectric points (pI), were calculated using ExPASy-ProtParam (https://web.expasy.org/protparam, accessed on 20 October 2025). Conserved domains were predicted using InterProScan with default parameters (https://www.ebi.ac.uk/interpro/, accessed on 20 October 2025). Homologous FlbB and FlbD proteins from representative fungi were retrieved from GenBank (https://www.ncbi.nlm.nih.gov/genbank, accessed on 20 October 2025), and sequence similarities were calculated using DNAman (version 6.0). Multiple sequence alignments were performed using ClustalX (version 2.1) with default parameters, and an unrooted phylogenetic tree was constructed in MEGA 7.0 using the neighbor-joining method with 1000 bootstrap replicates and pairwise deletion for gaps or missing data [27].

### 2.3. Generation and Verification of ΔAoflbB and ΔAoflbD Mutants

The Δ*AoflbB* and Δ*AoflbD* mutants were generated in the WT background via homologous gene replacement [28,29,30]. The coding sequences of *AoflbB* and *AoflbD*, together with their upstream and downstream flanking regions, were retrieved from the *A. oligospora* genome. Primers used for the amplification of homologous arms, the construction of replacement cassettes, diagnostic PCR, and real-time quantitative PCR (RT-qPCR) verification were designed using SnapGene (version 7.0.0), and they are listed in Table S1. For each target gene, upstream and downstream homologous fragments of approximately 2.0–2.5 kb were amplified via PCR, and the hygromycin B resistance cassette (*hph*) was amplified from pCSN44.

Replacement vectors were assembled via yeast-mediated homologous recombination [28,29]. The upstream homologous arm, *hph* cassette, downstream homologous arm, and linearized pRS426 vector were co-transformed into competent *S. cerevisiae* FY834 cells. Yeast transformants carrying recombinant plasmids were selected on SC-Ura medium and subsequently cultured in YPD medium for plasmid recovery. The complete replacement cassette was amplified from the recombinant plasmid and introduced into WT protoplasts using a CaCl_2_/PEG-mediated transformation method [30]. Transformants were recovered on the PDAS regeneration medium, consisting of PDA supplemented with 10 g/L molasses and 0.6 M sucrose and containing 200 μg/mL hygromycin B. Stable transformants were obtained through successive subculturing. Candidate mutants were initially screened via diagnostic PCR using genomic DNA as the template, and the loss of *AoflbB* or *AoflbD* transcripts was further verified via RT-qPCR.

### 2.4. Phenotypic Assay

The mycelial growth of WT, Δ*AoflbB*, and Δ*AoflbD* strains was compared on PDA, TYGA, and TG media [31]. Mycelial plugs of the same size were taken from actively growing colony margins and transferred to fresh plates. Colony diameters were recorded at 24 h intervals for 5 days, and radial growth rates were calculated. Each assay was performed with three biological replicates.

For cellular observations, sterile coverslips were inserted into culture plates before inoculation to allow young hyphae to extend along the glass surface. Nascent hyphae attached to coverslips were collected for fluorescence staining. Septa were visualized using 20 μg/mL of Calcofluor White (CFW; Sigma, St. Louis, MO, USA), nuclei were stained with 10 μg/mL of DAPI (Sigma, St. Louis, MO, USA) for 30 min, and endocytosis was monitored using 10 μg/mL of FM4-64 (Biotium, California, CA, USA) [32]. Samples were examined under a fluorescence microscope, and randomly selected hyphal cells were used to determine cell length, nuclear number, and FM4-64 internalization dynamics.

For ultrastructural observation, fresh hyphae grown on PDA for 5 days were harvested and immediately fixed [24]. After routine preparation for transmission electron microscopy, the cell wall morphology and intracellular vesicle-like structures were examined using a JEM-1400PLUS transmission electron microscope (TEM, JEOL, Tokyo, Japan).

### 2.5. Analysis of Sporulation and Spore Germination

For spore yield analysis, the WT, Δ*AoflbB*, and Δ*AoflbD* strains were cultured on corn meal–yeast extract (CMY) medium at 28 °C for 10–14 days [18,19,20]. Spores were harvested from each plate with 20 mL of sterile ddH_2_O, filtered to remove mycelial debris, before being counted using a hemocytometer.

For germination assays, spore suspensions were adjusted to the same concentration, and approximately 2 × 10^4^ spores were spread on MM plates. MM medium contained 10 g of glucose, 6 g of NaNO_3_, 1.52 g of KH_2_PO_4_, 0.52 g of KCl, 0.52 g of MgSO_4_·7H_2_O, 1 mL of trace element solution, and 20 g of agar per liter. After incubation at 28 °C, the germination rate was examined at 4, 8, and 12 h [33].

To observe the conidiophore morphology, strains were grown on PDA for 3 days. Mycelial blocks from colony margins were then transferred to WA plates and incubated at 28 °C for 12 h. Conidiophores were observed and photographed using a light microscope. All assays were performed with three biological replicates.

### 2.6. Analysis of Cell Wall Stress Response

Cell wall stress sensitivity was examined on TG medium supplemented with Congo red or SDS [21,24]. WT, Δ*AoflbB*, and Δ*AoflbD* strains were first grown on PDA at 28 °C for 5 days, and mycelial plugs of the same size were taken from actively growing colony margins. The strains were inoculated onto TG plates supplemented with Congo red (0.06, 0.09, or 0.12 mg/mL) or SDS (0.01%, 0.02%, or 0.03%). After incubation at 28 °C for 5 days, colony diameters were measured, and relative growth inhibition (RGI) was calculated using untreated TG plates as controls [31]. Each treatment was performed with three biological replicates.

### 2.7. Trap Formation, Pathogenicity, and Extracellular Protease Assays

Trap formation was induced on WA plates for the WT, Δ*AoflbB*, and Δ*AoflbD* strains. Approximately 2 × 10^4^ spores were evenly spread onto each WA plate. After incubation at 28 °C for 3 days, about 400 *C. elegans* nematodes were added to each plate. Trap formation and nematode survival were examined under a light microscope at 12, 24, 36, and 48 h after induction. Trap numbers were counted, and nematode mortality was calculated as the ratio of dead nematodes to the total number of nematodes counted. The natural mortality of nematodes on WA plates was included as the control. Three biological replicates were performed for each strain. Extracellular protease activity was evaluated via a skim milk plate assay, as described previously [34].

### 2.8. Gene Expression Analysis

For RT-qPCR analysis, the WT, Δ*AoflbB*, and Δ*AoflbD* strains were cultured on PDA plates overlaid with sterile cellophane at 28 °C for 5 days. The mycelia were collected, immediately frozen in liquid nitrogen, and ground into powder. Total RNA was extracted using TRIzol reagent, and first-strand cDNA was synthesized using the PrimeScript RT Reagent Kit (Takara, Shiga, Japan). RT-qPCR was performed to determine the transcript levels of target genes and those related to sporulation and cell wall biosynthesis. For sporulation-related gene expression analysis, fungal samples were collected at 3, 5, and 7 days of spore development. The *β-tubulin* gene (*AOL_s0076g640*) was used as the internal reference, and relative transcript levels were calculated using the 2^−ΔΔCt^ method [35]. Primer sequences are listed in Supplementary Table S1. Each assay was performed with three biological replicates.

### 2.9. LC-MS-Based Analysis of Extracellular Metabolites

WT, Δ*AoflbB*, and Δ*AoflbD* strains were activated on PDA at 28 °C for 5 days before liquid fermentation. Mycelial plugs with comparable colony growth were transferred into PDB and incubated for 7 days at 28 °C with shaking at 180 rpm. The cultures were then filtered to separate fungal biomass from the fermentation broth. The mycelial dry weight was recorded for normalization, and the corresponding culture filtrates were used for metabolite extraction.

For extraction, each culture filtrate was mixed with an equal volume of ethyl acetate. After phase separation, the organic phase was collected, concentrated to dryness, and reconstituted in chromatographic-grade methanol [25,26]. The final sample volume was adjusted according to fungal biomass before performing LC-MS analysis. LC-MS analysis was performed using a UHPLC system coupled to a high-resolution Orbitrap mass spectrometer (Thermo Fisher Scientific, Bremen, Germany). Chromatographic separation was carried out on a reversed-phase column (2.1 mm × 100 mm, 1.8 μm). The mobile phase consisted of solvent A, water containing ammonium acetate and acetic acid, and solvent B, acetonitrile. Samples were maintained at low temperature in the autosampler, and the injection volume was 2 μL. Data were acquired in both positive and negative electrospray ionization modes. Full MS and MS/MS spectra were collected under data-dependent acquisition with high-resolution settings. Mass spectrometry data were processed using Compound Discoverer 3.0, including feature detection, retention-time alignment, peak-area integration, and comparative analysis among strains. Metabolite features were annotated according to accurate mass, retention time, and MS/MS fragmentation information. Three independent biological replicates were prepared for each strain [26].

### 2.10. Transcriptomic Analysis

WT and Δ*AoflbD* strains were incubated on PDA plates at 28 °C for 5 days. Mycelia collected before nematode exposure were used as the uninduced control sample (0 h). For nematode induction, approximately 400 *C. elegans* nematodes were added to the remaining cultures, and mycelial samples were harvested 12 h later. All samples were immediately frozen in liquid nitrogen. RNA isolation, library preparation, and high-throughput sequencing were performed by Gene Denovo Biotechnology Co., Ltd. (Guangzhou, China). RNA quality was evaluated by agarose gel electrophoresis, spectrophotometric analysis, Qubit fluorometric quantification, and Agilent 2100 Bioanalyzer analysis (Agilent Technologies, Santa Clara, CA, USA). Three biological replicates were prepared for each treatment. Raw reads were filtered using fastp (version 0.18.0), and rRNA-derived reads were removed by Bowtie2 (version 2.4.4) alignment. The retained clean reads were mapped to the *A. oligospora* reference genome GCF_000225545.1_AOL using HISAT2 (version 2.1.0). Transcript reconstruction and gene expression quantification were performed using StringTie (version 1.3.4) and Salmon (version 1.10.3), respectively, and gene expression levels were calculated as raw read counts and FPKM values. Genes with |log_2_ fold change| ≥ 1 and FDR < 0.05 were defined as differentially expressed genes (DEGs). GO annotation and KEGG pathway enrichment analyses were performed for the DEGs. Transcriptomic data analysis and visualization were conducted using the OmicShare bioinformatics platform (https://www.omicshare.com, accessed on 16 March 2026) [36], following a previously published transcriptomic study of *A. oligospora* [37].

### 2.11. Statistical Analysis

Quantitative results are expressed as the mean ± SD of three independent biological replicates. Statistical calculations were carried out using GraphPad Prism 9.0. For comparisons among multiple groups, one-way ANOVA was used, followed by Tukey’s test for multiple comparisons. Differences were considered statistically significant when *p* < 0.05.

## 3. Results

### 3.1. Bioinformatic Analysis of AoFlbB and AoFlbD and Verification of Deletion Mutants

**Figure 1:**
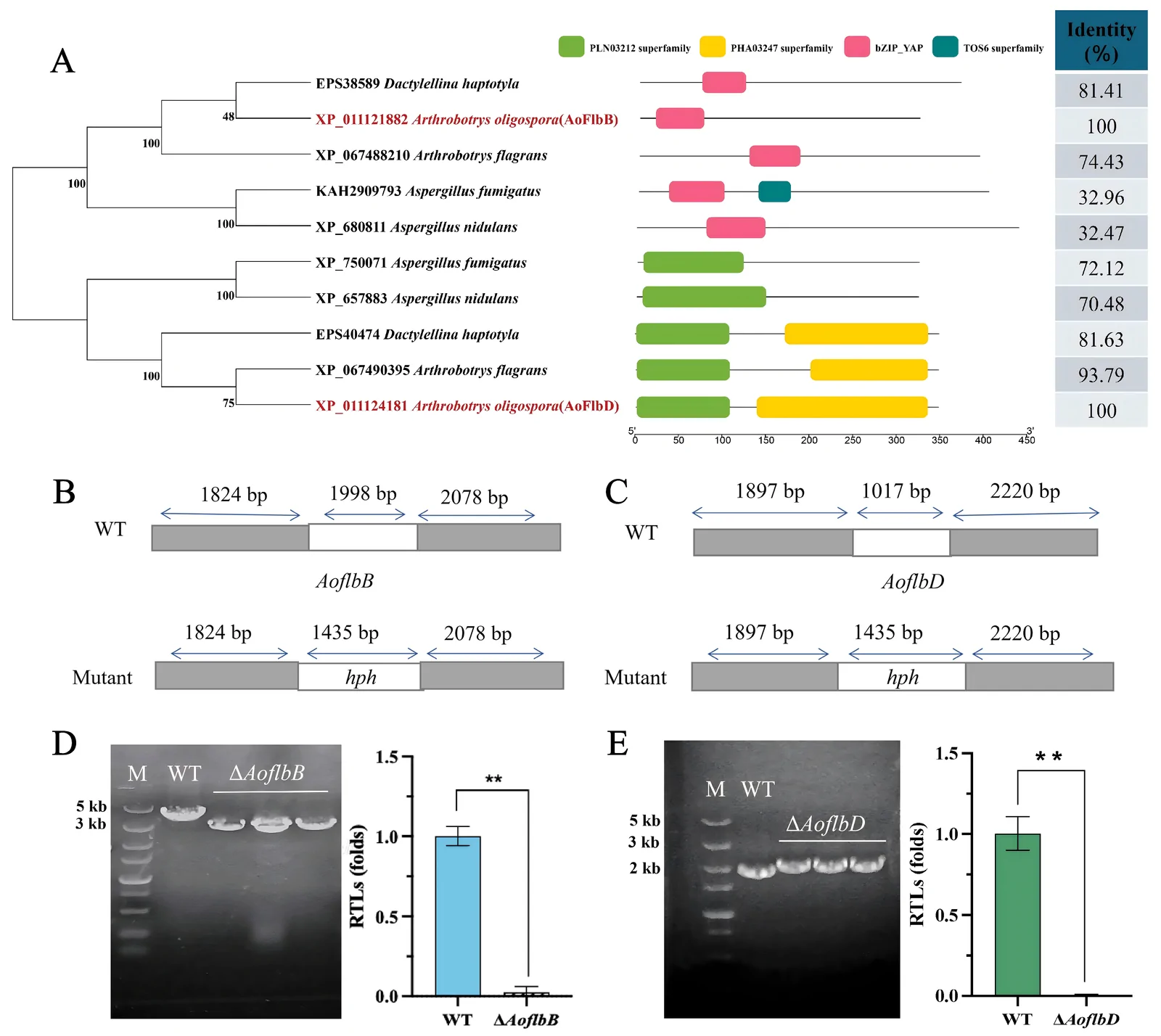
Phylogenetic analysis of AoFlbB and AoFlbD and verification of deletion mutants in *A. oligospora*. (**A**) Phylogenetic analysis of AoFlbB, AoFlbD, and their homologs from different fungi, together with conserved domain prediction. The phylogenetic tree was constructed using MEGA 7.0. (**B**,**C**) Schematic diagrams of the homologous replacement strategies used to delete *AoflbB* (**B**) and *AoflbD* (**C**). (**D**,**E**) Verification of the Δ*AoflbB* (**D**) and Δ*AoflbD* (**E**) mutants via PCR and RT-qPCR. RTL, relative transcription level. For quantitative panels, data are sourced from three independent biological replicates. Asterisks indicate statistical significance: **, *p* < 0.01.

AoFlbB and AoFlbD were identified as homologs of FlbB and FlbD, respectively, in *A. oligospora*. AoFlbB contains 312 amino acids, with a predicted pI of 5.59 and a molecular mass of 33.99 kDa, whereas AoFlbD contains 338 amino acids, with a predicted pI of 10.12 and a molecular mass of 38.10 kDa. Conserved domain analysis showed that AoFlbB contains a bZIP_YAP domain, whereas AoFlbD was predicted to be a MYB-like DNA-binding protein containing the PLN03212 and PHA03247 superfamily domains. Phylogenetic and pairwise identity analyses further supported the classification of AoFlbB and AoFlbD as FlbB- and FlbD-type homologs, respectively. AoFlbB shared 81.41% and 74.43% identity with FlbB homologs from the NT fungi *D*. *haptotyla* and *A*. *flagrans*, respectively, but they showed lower identity with those from *A*. *fumigatus* and *A*. *nidulans* (32.96% and 32.47%, respectively). AoFlbD shared 93.79% and 81.63% identity with homologs from *A*. *flagrans* and *D*. *haptotyla*, respectively, and 72.12% and 70.48% identity with those from *A*. *fumigatus* and *A*. *nidulans*, respectively (Figure 1A).

To investigate the functions of *AoflbB* and *AoflbD*, deletion mutants were generated via homologous replacement. The replacement strategies for *AoflbB* and *AoflbD* are shown in Figure 1B,C, respectively. Candidate Δ*AoflbB* and Δ*AoflbD* transformants were screened via diagnostic PCR, which produced the expected fragments for homologous replacement at the target loci (Figure 1D,E). RT-qPCR analysis further showed that the transcript levels of *AoflbB* and *AoflbD* were almost undetectable in the corresponding mutants, confirming the successful generation of the deletion mutants (Figure 1D,E).

### 3.2. AoFlbB and AoFlbD Positively Regulate Sporulation

**Figure 2:**
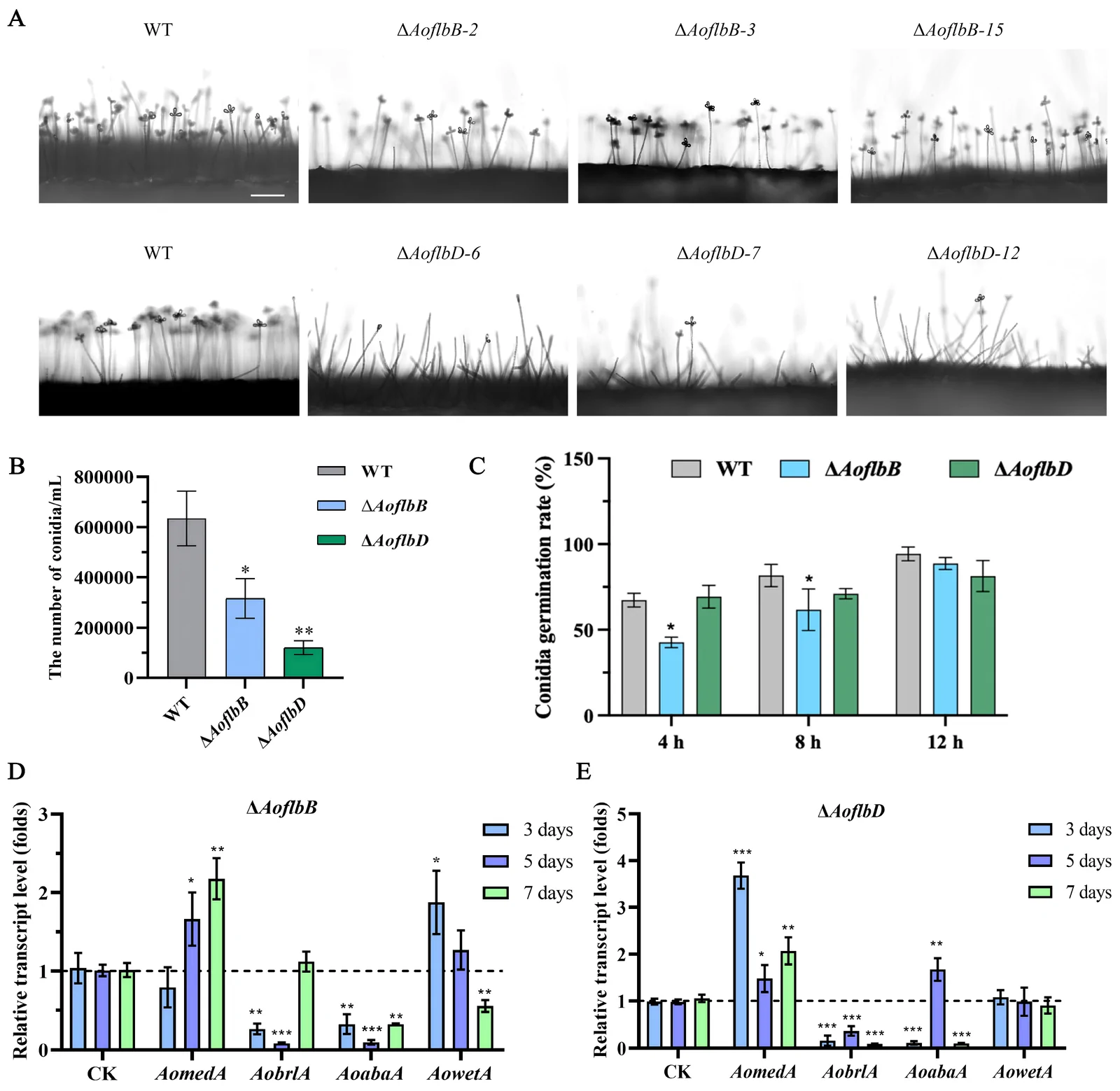
Effects of *AoflbB* and *AoflbD* deletion on sporulation and spore germination in *A. oligospora*. (**A**) Conidiophore morphology of WT, Δ*AoflbB*, and Δ*AoflbD* strains. Scale bar, 100 μm. (**B**) Quantification of spore production in WT and mutant strains. (**C**) Spore germination rates of WT, Δ*AoflbB*, and Δ*AoflbD* strains at 4, 8, and 12 h. (**D**) RT-qPCR analysis of sporulation-related genes in Δ*AoflbB*. (**E**) RT-qPCR analysis of sporulation-related genes in Δ*AoflbD*. For quantitative panels, data are from three independent biological replicates. Asterisks indicate statistical significance: *, *p* < 0.05; **, *p* < 0.01; ***, *p* < 0.001.

Sporulation was markedly impaired via the deletion of either *AoflbB* or *AoflbD*. WT formed typical conidiophores and produced abundant spores, whereas both mutants showed defective sporulation, with sparse spore attachment or severely reduced spore formation (Figure 2A). Quantitative analysis showed that spore yield in Δ*AoflbB* decreased to approximately 60% of the WT level, while that in Δ*AoflbD* decreased to approximately 20% of the WT level, corresponding to about 1.7- and 5-fold reductions, respectively (Figure 2B). Spore germination was also affected differently in the two mutants. Compared with WT, the germination rate of Δ*AoflbB* decreased to approximately two-thirds of the WT level at 4 h and about three-quarters of the WT level at 8 h, whereas Δ*AoflbD* showed no significant difference from WT during the examined period (Figure 2C). Several sporulation-related genes showed disrupted temporal expression in the mutants during spore development (Figure 2D,E). In Δ*AoflbB*, *AobrlA* was reduced at 3 and 5 days, *AoabaA* was reduced at all examined time points, *AomedA* was induced at 5 and 7 days, and *AowetA* was upregulated at 3 days but downregulated at 7 days. In Δ*AoflbD*, *AomedA* was strongly induced at 3, 5, and 7 days, whereas *AobrlA* was consistently reduced throughout development. *AoabaA* decreased at 3 and 7 days but increased at 5 days, while *AowetA* showed no obvious change. These results indicate that AoFlbB and AoFlbD are required for normal spore production and the proper temporal regulation of sporulation-related genes, with AoFlbB additionally contributing to early spore germination.

### 3.3. AoFlbB and AoFlbD Negatively Regulate Trap Formation and Pathogenicity

**Figure 3:**
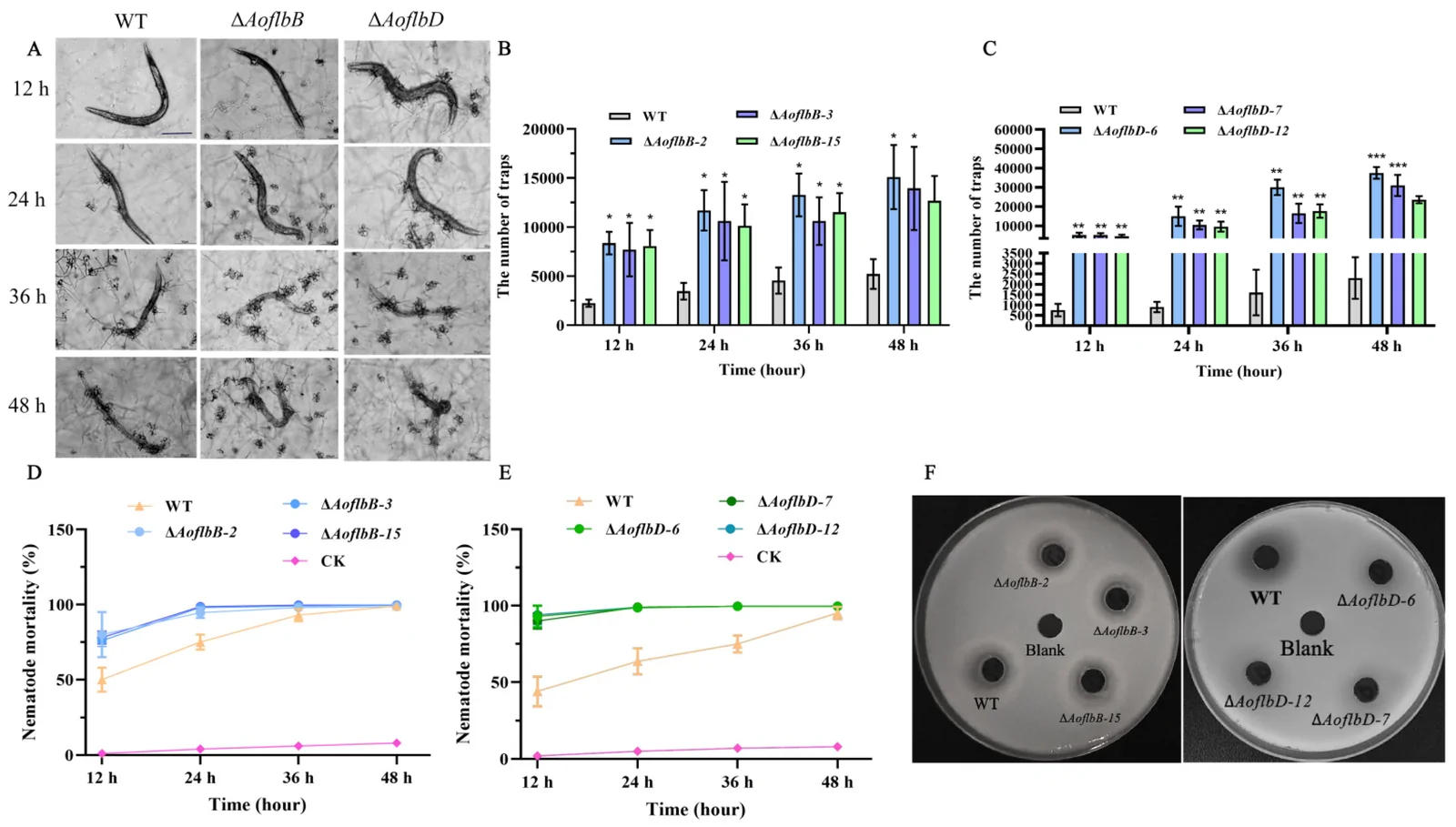
Deletion of *AoflbB* or *AoflbD* enhances trap formation and pathogenicity in *A. oligospora*. (**A**) Trap formation for WT, Δ*AoflbB*, and Δ*AoflbD* strains at 12, 24, 36, and 48 h after nematode induction. Scale bar, 100 μm. (**B**) Quantification of trap numbers per plate in WT and Δ*AoflbB* strains at different induction times. (**C**) Quantification of trap numbers per plate in WT and Δ*AoflbD* strains at different induction times. (**D**,**E**) Nematode mortality in WT and Δ*AoflbB* (**D**) and Δ*AoflbD* (**E**) cultures at different induction times. (**F**) Extracellular protease activity of WT and mutant strains assessed via skim milk hydrolysis assay. For quantitative panels, data are sourced from three independent biological replicates. Asterisks indicate significant differences compared with WT at the same time point: *, *p* < 0.05; **, *p* < 0.01; ***, *p* < 0.001.

Nematode induction revealed significant differences in trap formation and pathogenicity between the WT and mutants. From 12 to 48 h, both Δ*AoflbB* and Δ*AoflbD* produced markedly more traps than WT (Figure 3A–C). Nematode mortality was also higher in the mutants, particularly at the early induction stages of 12 and 24 h (Figure 3D,E). In Δ*AoflbD*, mortality reached approximately 90% at 12 h, whereas WT showed only about 40% mortality at the same time point (Figure 3E). These results indicate that loss of either *AoflbB* or *AoflbD* enhances trap formation and pathogenicity in *A. oligospora*. Extracellular protease activity was then examined using a skim milk hydrolysis assay. Δ*AoflbB* produced hydrolysis zones comparable to those of WT, whereas Δ*AoflbD* produced smaller hydrolysis zones (Figure 3F). Thus, the enhanced pathogenicity of the mutants was not associated with increased extracellular protease activity. Together, these data suggest that AoFlbB and AoFlbD negatively regulate trap formation and pathogenicity, while exerting different effects on extracellular protease activity.

### 3.4. AoFlbB and AoFlbD Regulate Hyphal Cell Morphology and Nuclear Number

**Figure 4:**
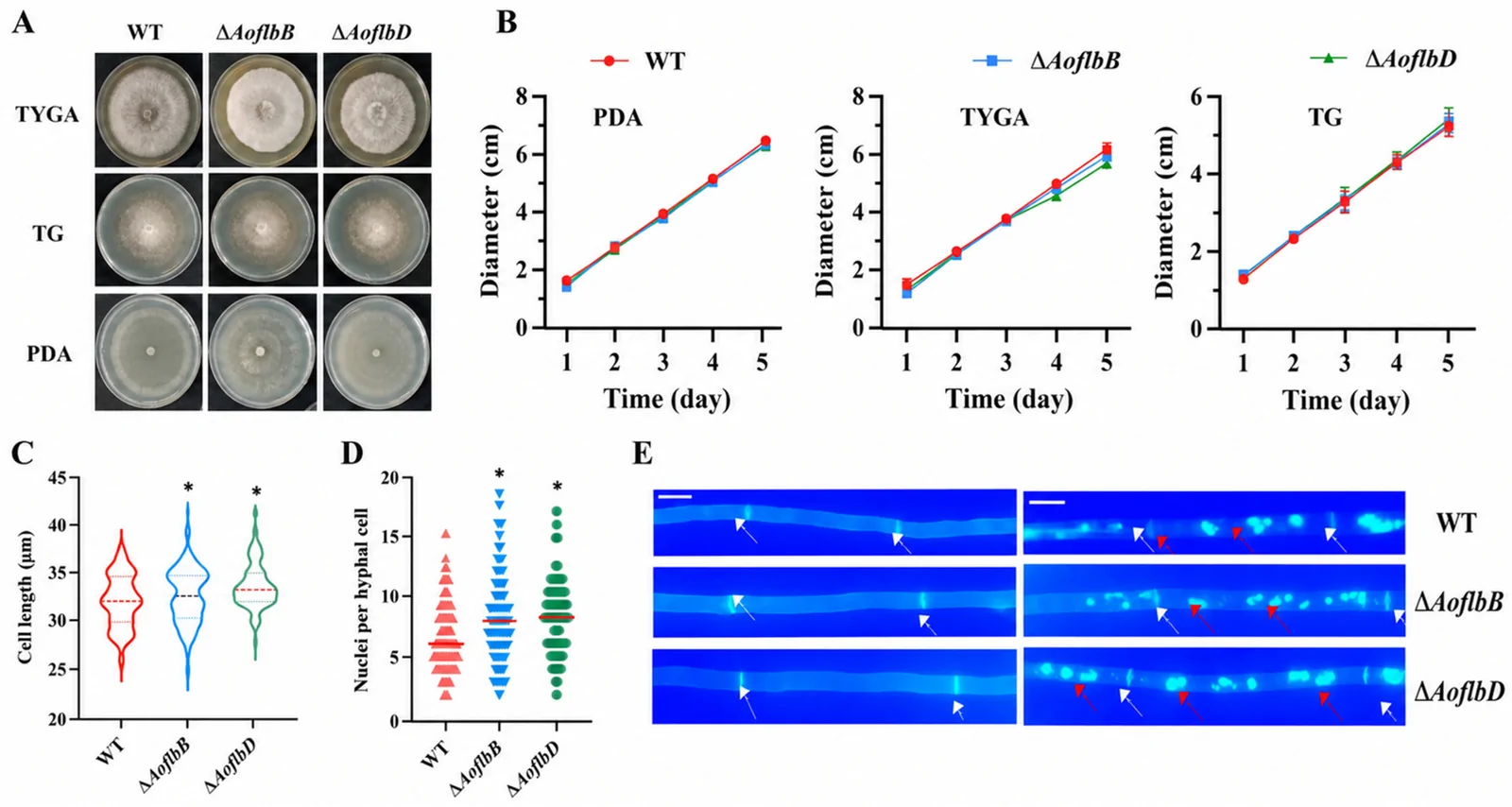
Effects of *AoflbB* and *AoflbD* deletion on radial growth, hyphal morphology, and nuclear number in *A. oligospora*. (**A**) Colony morphologies of WT, Δ*AoflbB*, and Δ*AoflbD* strains cultured on TYGA, TG, and PDA media. (**B**) Colony diameters of WT and mutant strains measured over 5 days on TYGA, TG, and PDA media. (**C**) Violin plots showing hyphal cell length in WT and mutant strains. (**D**) Quantification of the number of nuclei per hyphal cell in WT and mutant strains. (**E**) CFW and DAPI staining showing septation patterns and nuclear distribution in WT and mutant hyphae. **Left**, CFW-stained septa; **right**, DAPI-stained nuclei. White arrows indicate septa, and red arrows indicate nuclei. Scale bar, 5 μm. For quantitative panels, data are sourced from three independent biological replicates. Asterisks indicate statistical significance: *, *p* < 0.05.

WT, Δ*AoflbB*, and Δ*AoflbD* mutant strains showed comparable colony morphology and radial growth on TYGA, TG, and PDA media over 5 days (Figure 4A,B), indicating that the loss of *AoflbB* or *AoflbD* had little effect on colony expansion. Hyphal septation and nuclear distribution were then examined via CFW and DAPI staining. Despite similar radial growth, both mutants showed increased hyphal cell length and a higher number of nuclei per hyphal cell compared with WT (Figure 4C–E). These results indicate that AoFlbB and AoFlbD are dispensable for radial colony growth but are required for normal hyphal cell morphology and nuclear distribution in *A. oligospora*.

### 3.5. AoFlbB and AoFlbD Are Involved in Cell Wall Stress Responses and Membrane-Associated Cellular Processes

**Figure 5:**
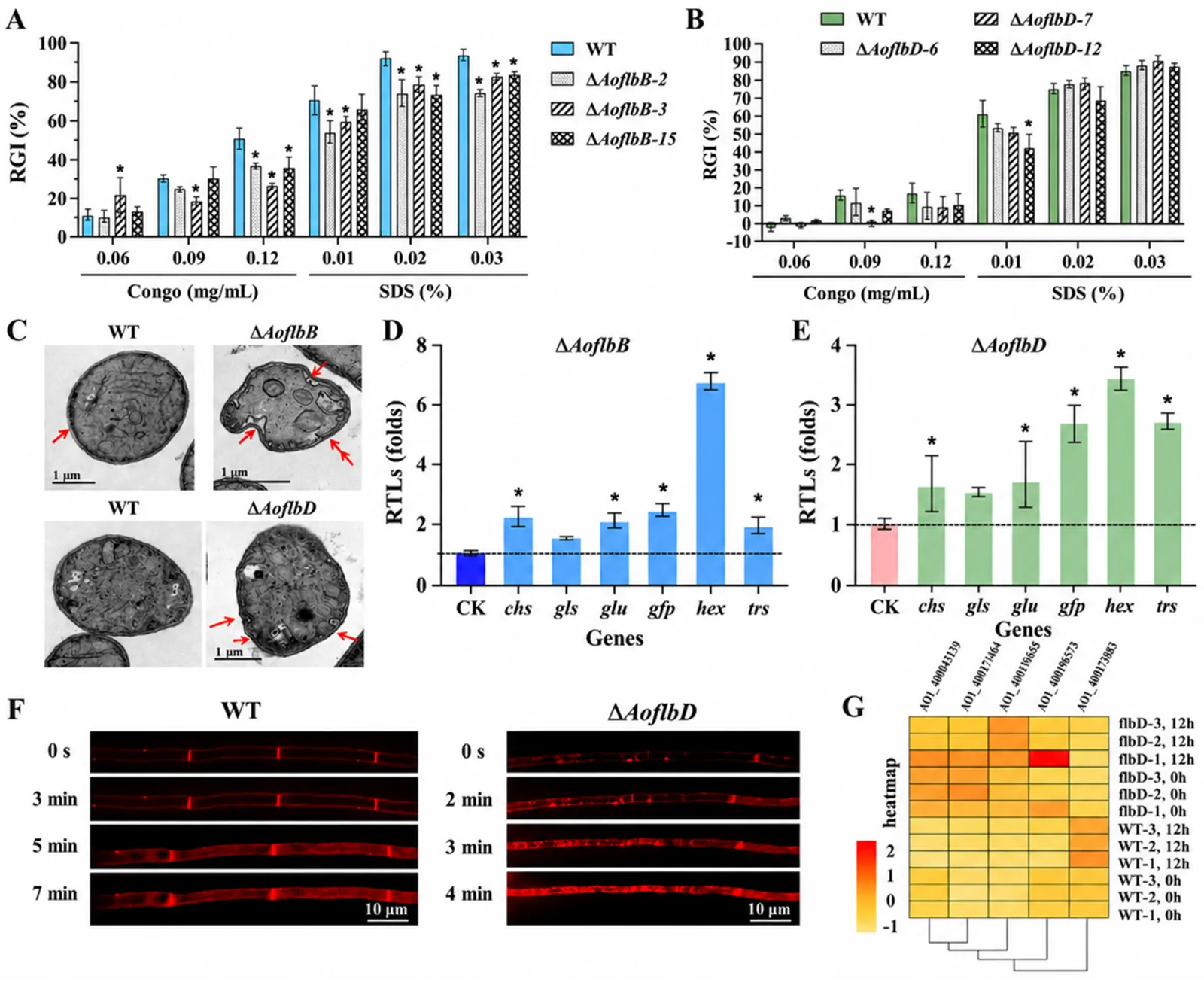
Deletion of *AoflbB* or *AoflbD* alters cell wall stress responses, cell wall biosynthesis gene expression, and endocytosis in *A. oligospora*. (**A**,**B**) RGI values of WT, Δ*AoflbB* (**A**), and Δ*AoflbD* (**B**) strains under Congo red and SDS. (**C**) TEM images showing cell wall morphology and intracellular vesicle-like structures in WT and mutant strains. Red arrows indicate cell wall abnormalities or vesicle-like structures. Scale bar, 1 μm. (**D**,**E**) RT-qPCR analysis of cell wall-related genes in Δ*AoflbB* (**D**) and Δ*AoflbD* (**E**) mutant strains. (**F**) Representative FM4-64 staining images showing membrane internalization in WT and Δ*AoflbD* hyphae. Scale bar, 10 μm. (**G**) Heatmap showing the expression patterns of selected membrane- and cell wall-associated genes in WT and Δ*AoflbD* samples. For quantitative panels, data are from three independent biological replicates. Asterisks indicate statistical significance: *, *p* < 0.05.

Cell wall stress assays were performed on TG medium supplemented with Congo red or SDS. Compared with WT, Δ*AoflbB* showed reduced RGI when treated with Congo red (0.12 mg/mL) and SDS (0.02% and 0.03%) (Figure 5A and Figure S1A). Δ*AoflbD* also showed lower RGI when treated with 0.09 mg/mL of Congo red, whereas its response to treatment with SDS was only weakly affected (Figure 5B and Figure S1B). These results indicate that the deletion of *AoflbB* broadly increases resistance to cell wall-perturbing agents, whereas *AoflbD* deletion mainly affects the response to Congo red. Ultrastructural observation further revealed distinct cellular changes in the two mutants. TEM showed irregular cell wall wrinkling in Δ*AoflbB*, whereas obvious accumulation of vesicle-like intracellular structures was observed in Δ*AoflbD* but not in Δ*AoflbB* (Figure 5C). RT-qPCR analysis showed that *chs*, *glu*, *gfp*, *hex*, and *trs* were significantly upregulated in both mutants, whereas gls showed no significant change. Among these genes, hex displayed the strongest increase in transcript abundance (Figure 5D,E). The specific accumulation of vesicle-like structures in Δ*AoflbD* prompted further examination of membrane trafficking in this mutant using FM4-64 staining. Intracellular fluorescence appeared earlier in Δ*AoflbD* than in WT, suggesting the occurrence of altered endocytic trafficking (Figure 5F). Transcriptome-based clustering further showed induction-dependent changes in selected membrane- and cell wall-associated genes in ΔAoflbD, with several genes displaying higher relative expression at 12 h after nematode induction (Figure 5G).

### 3.6. AoFlbB and AoFlbD Play a Crucial Role in Extracellular Metabolite Profiles

**Figure 6:**
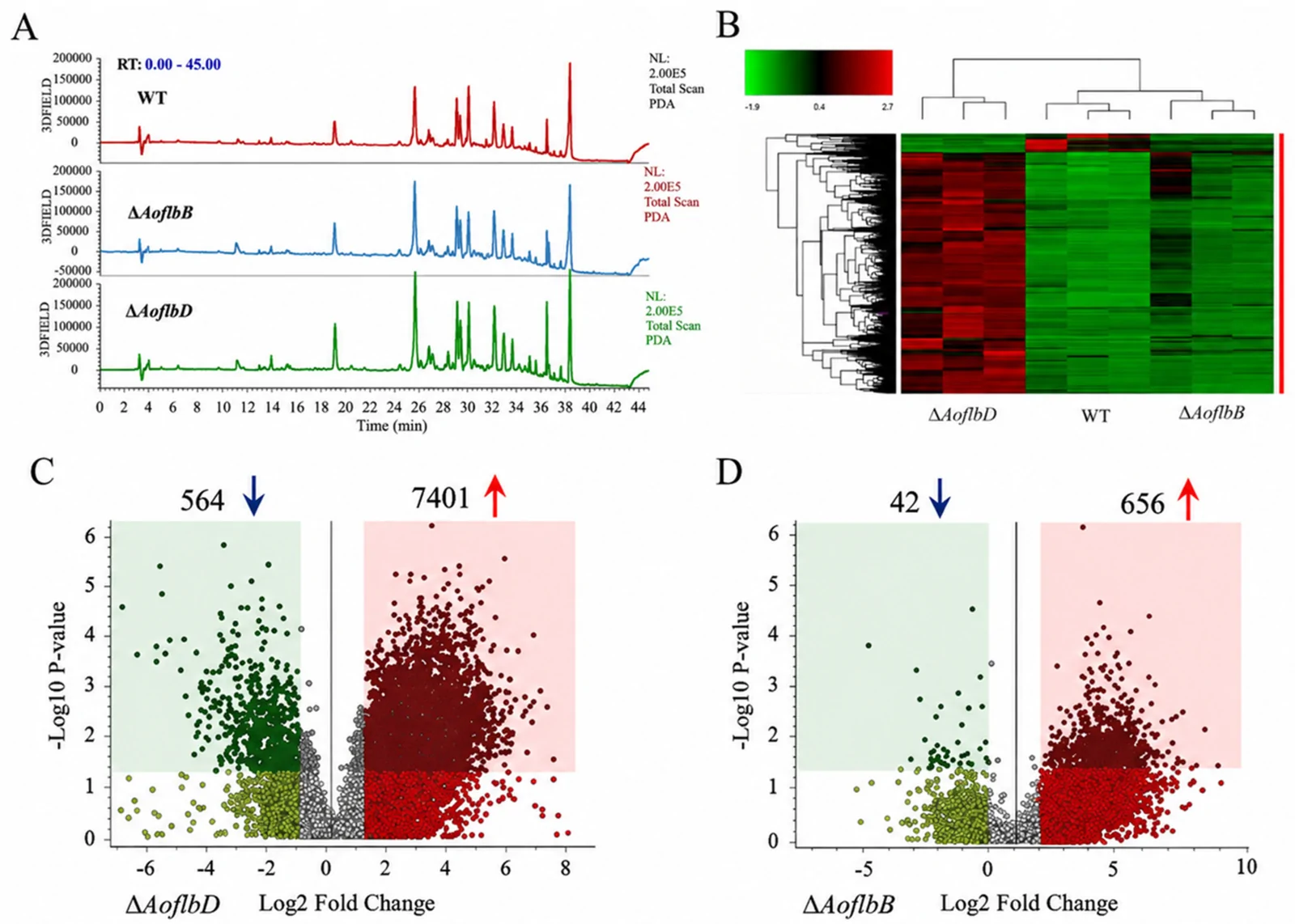
Deletion of *AoflbB* or *AoflbD* alters extracellular metabolite profiles in *A. oligospora*. (**A**) Representative chromatograms of extracellular metabolites extracted from fermentation supernatants of WT, Δ*AoflbB*, and Δ*AoflbD* strains. (**B**) Hierarchical clustering heatmap showing differences in extracellular metabolite profiles among WT, Δ*AoflbB*, and Δ*AoflbD* strains. (**C**) Volcano plot showing differential metabolic features between Δ*AoflbD* and WT. (**D**) Volcano plot showing differential metabolic features between Δ*AoflbB* and WT. The blue arrow in (**C**,**D**) indicates the downregulated metabolites, and the red arrow indicate the upregulated metabolites.

LC-MS profiling of fermentation supernatants revealed changes in the extracellular metabolite profiles of both mutants. Total ion chromatograms showed distinct peak-intensity patterns among the WT, Δ*AoflbB*, and Δ*AoflbD* strains (Figure 6A), and hierarchical clustering based on metabolite features separated the three strains into different groups (Figure 6B). Differential feature analysis identified 7401 increased and 564 decreased features in Δ*AoflbD* and 656 increased and 42 decreased features in Δ*AoflbB*, relative to WT (Figure 6C,D). These results indicate that extracellular metabolite profiles were affected in both mutants, with a much stronger effect noted in Δ*AoflbD*.

### 3.7. AoFlbD Regulates Transcriptional Reprogramming During Nematode Induction

**Figure 7:**
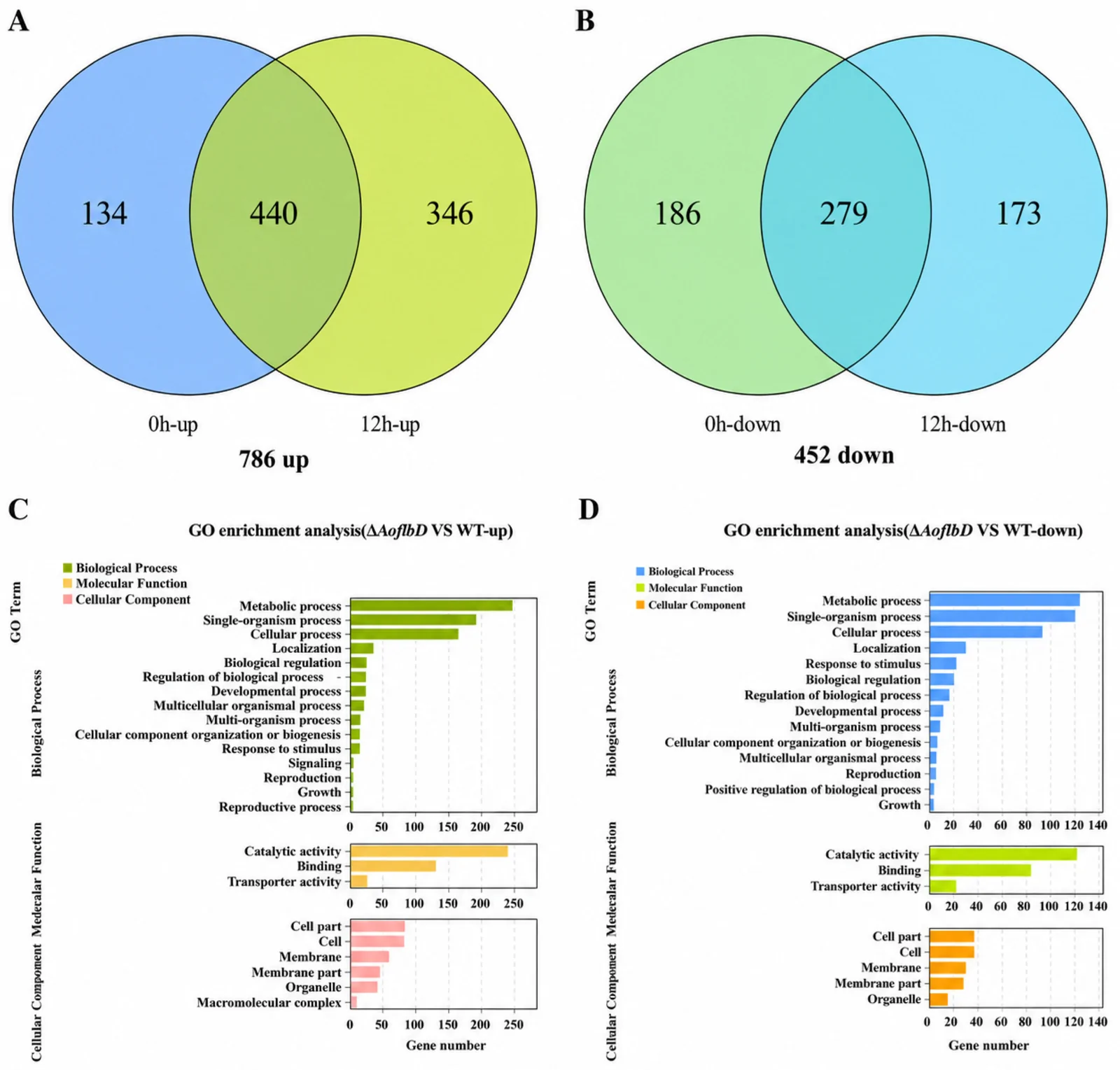
AoFlbD regulates transcriptional reprogramming during nematode induction in *A. oligospora*. (**A**,**B**) Venn analysis of up- (**A**) and downregulated (**B**) DEGs identified in Δ*AoflbD* relative to WT at 0 h and 12 h after nematode induction. (**C**,**D**) GO enrichment analysis of up- (**C**) and downregulated (**D**) DEGs in Δ*AoflbD* relative to WT after 12 h of nematode induction.

RNA-seq was used to assess how *AoflbD* deletion affects the transcriptional response to nematode induction. Hierarchical clustering separated WT and Δ*AoflbD* samples into distinct groups, with biological replicates clustering together, indicating the high reproducibility of the transcriptomic dataset (Figure S2). The DEG list is provided in Supplementary Table S2. Venn analysis identified 786 genes that were upregulated in Δ*AoflbD* relative to WT at 12 h, including 440 genes shared with the 0 h comparison and 346 genes specifically upregulated at 12 h. In addition, 452 genes were downregulated at 12 h, including 279 shared genes and 173 genes specifically downregulated at 12 h (Figure 7A,B). These results indicate that the deletion of *AoflbD* substantially altered the transcriptional response of *A. oligospora* during nematode induction. The KEGG enrichment analysis showed that the DEGs were mainly associated with metabolism-related pathways, including amino acid, carbohydrate, lipid, secondary metabolite, carotenoid, and unsaturated fatty acid biosynthesis, as well as glycolysis/gluconeogenesis (Figure S3A,B). GO enrichment analysis further identified terms related to metabolic process, catalytic activity, membrane components, and transmembrane transport (Figure 7C,D). Together, these results suggest that AoFlbD contributes to transcriptional reprogramming during nematode induction, particularly in pathways associated with metabolism and membrane-related cellular processes.

## 4. Discussion

Flb proteins are conserved upstream regulators of asexual development in filamentous fungi [38,39]. In *A. nidulans*, FlbB and FlbD act upstream of the central *brlA*–*abaA*–*wetA* cascade and are required for initiating asexual sporulation [15,16,17]. Although Flb proteins have been studied extensively in *Aspergillus* species, their functions in NT fungi remain poorly defined. In this study, we identified two Flb homologs, AoFlbB and AoFlbD, in *A. oligospora*. Both genes were required for normal sporulation, whereas their deletion enhanced trap formation and pathogenicity. In addition to these shared phenotypic traits, the two mutants showed partially distinct cellular and molecular changes. Δ*AoflbB* displayed reduced early spore germination and more evident cell wall distortion, whereas Δ*AoflbD* showed stronger pathogenicity, vesicle-like accumulation, earlier intracellular FM4-64 fluorescence, broader changes in extracellular metabolite profiles, and extensive transcriptional reprogramming during nematode induction. Taken together, these findings suggest that Flb-type regulators connect asexual development with predatory differentiation in an NT fungus.

The conserved developmental functions of AoFlbB and AoFlbD were most clearly reflected in sporulation. Both Δ*AoflbB* and Δ*AoflbD* mutants produced markedly fewer spores (Figure 2), indicating that the two act as positive regulators of asexual development in *A. oligospora*. This phenotype is consistent with the established role of FlbB and FlbD in *A. nidulans*, where they promote *brlA* activation and initiate the central developmental program controlling asexual sporulation [17]. However, the expression patterns of several sporulation-associated regulators, including *AomedA*, *AobrlA*, *AoabaA*, and *AowetA*, were not uniformly reduced in the mutants (Figure 2). Instead, these genes showed gene-specific, mutant-specific, and stage-dependent responses. This suggests that AoFlbB and AoFlbD do not simply activate all components of the sporulation pathway through a linear cascade. Rather, they may contribute to the timing and balance of the sporulation regulatory network through partially distinct routes. The selective reduction in early spore germination in Δ*AoflbB* further suggests that AoFlbB may also influence spore maturation or spore quality, a function that was not equally noted in AoFlbD. Previous studies in *A. nidulans* have demonstrated FlbB and FlbD as upstream regulators of the BrlA–AbaA–WetA developmental cascade. However, whether AoFlbB and AoFlbD regulate this pathway in the same manner in *A. oligospora* remains to be experimentally determined.

In contrast to their positive roles in sporulation, the deletion of either *AoflbB* or *AoflbD* increased trap formation and pathogenicity (Figure 3). This result suggests that asexual development and predatory differentiation are closely connected in *A*. *oligospora*, but these two processes are not always promoted in the same direction. Trap development is a defining feature of NT fungi and depends on prey-derived cues and extensive morphogenetic changes [6,7]. A similar relationship between sporulation and trap formation has been reported in several regulatory mutants of *A*. *oligospora*. For example, the deletion of central sporulation regulators, especially *AobrlA* and *AoabaA*, severely impaired spore production but increased trap formation during nematode induction [19]. A comparable trend was reported for Δ*Aossk1*, in which reduced spore production and lower expression of sporulation-related genes were accompanied by increased trap formation and predation efficiency [21]. In addition, AoCryA provides another relevant example: under dark conditions, the deletion of *AocryA* reduced spore production and the expression of several sporulation-related genes, while enhancing trap formation and nematode-trapping ability [33]. These studies suggest that some regulatory pathways in *A*. *oligospora* may shift development away from sporulation and toward predatory differentiation. Our results extend this relationship to upstream Flb-type regulators. Thus, AoFlbB and AoFlbD appear to promote sporulation while restraining trap formation and pathogenicity under the tested conditions. These results broaden the known roles of Flb proteins beyond asexual development and suggest that, in NT fungi, Flb-type regulators may also contribute to coordinating reproductive development with trap-mediated predation.

The enhanced pathogenicity of the mutants was not accompanied by increased extracellular protease activity. Extracellular serine proteases are known to contribute to nematode degradation after capture and are important pathogenicity-related factors in *A. oligospora* and other NT fungi [34]. However, Δ*AoflbB* showed proteolytic activity comparable to WT, whereas Δ*AoflbD* formed smaller hydrolysis zones than WT despite showing significantly higher pathogenicity (Figure 3). These results indicate that increased pathogenicity in the mutants cannot be attributed solely to increased extracellular protease activity. Instead, other factors, such as increased trap number, trap maturation, adhesive capacity, or infection dynamics, are likely to contribute to the enhanced pathogenic phenotype [40]. The skim milk assay reflects general extracellular protease activity, but it does not exclude the possibility that specific nematode-induced proteases or other secreted factors may be differentially regulated during direct prey interaction. Therefore, the current data support the conclusion that overall extracellular protease activity is not the main explanation for the increased pathogenicity of Δ*AoflbB* and Δ*AoflbD*.

At the cellular level, AoFlbB and AoFlbD also influenced hyphal organization. Although colony morphology and radial growth were largely unchanged on different media, both mutants formed elongated hyphal compartments and contained more nuclei per hyphal cell (Figure 4). These findings indicate that AoFlbB and AoFlbD are not required for colony expansion under the tested conditions, but they are involved in maintaining normal hyphal cell morphology, septation-associated organization, and nuclear distribution. In *A*. *oligospora*, the cell wall integrity-related regulators AoBck1 and AoMkk1 have been shown to affect vegetative growth, cell nucleus development, stress resistance, and the expression of cell wall biosynthesis-related genes [31]. The Slt2 MAP kinase has also been linked to sporulation, trap formation, pathogenicity, and cell wall-related phenotypes in NT fungi [41]. The mutants also showed modified responses to cell wall-perturbing agents and increased expression of several cell wall biosynthesis-related genes. However, the reduced RGI recorded for treatment with Congo red or SDS should not simply be interpreted as indicating a uniformly stronger cell wall. Together with the irregular cell wall wrinkling observed in ΔAoflbB, these results are more consistent with cell wall remodeling or compensatory changes in cell wall organization. Thus, AoFlbB may contribute to maintaining normal cell wall organization.

AoFlbD appeared to play an additional role in membrane-associated cellular processes. TEM observation revealed increased vesicle-like intracellular structures in Δ*AoflbD*, and FM4-64 staining showed earlier intracellular fluorescence than in WT (Figure 5). FM4-64 has been widely used to monitor endocytosis and vesicle trafficking in living fungal hyphae [32]. Therefore, these observations are consistent with altered endocytic trafficking in Δ*AoflbD*. Previous studies have shown that membrane curvature, endocytosis, vesicle trafficking, and vacuole-associated processes are closely linked to trap formation, pathogenicity, stress responses, and secondary metabolism in *A. oligospora* [22,23,24]. Transcriptome-based clustering of selected membrane- and cell wall-associated genes further showed changed expression patterns in Δ*AoflbD* during nematode induction, with several genes displaying higher relative expression at 12 h (Figure 5). These observations suggest that AoFlbD may participate in cellular remodeling associated with membrane trafficking and cell wall-related responses. Nevertheless, the current FM4-64 and TEM observations are qualitative, and they do not distinguish increased endocytic flux from changes in membrane composition, vesicle maturation, recycling, or vacuolar transport. Performing further live-cell imaging and quantitative endocytosis assays will be necessary to clarify how AoFlbD affects membrane trafficking during predatory development.

Metabolic remodeling represents another major difference between AoFlbB and AoFlbD. LC-MS-based profiling of fermentation supernatants revealed changes in the extracellular metabolite profiles of both mutants, with a much stronger effect in Δ*AoflbD* than in Δ*AoflbB* (Figure 6). This pattern was consistent with the RNA-seq data from Δ*AoflbD* recorded during nematode induction. The affected pathways included amino acid metabolism, carbohydrate metabolism, lipid metabolism, secondary metabolite biosynthesis, carotenoid biosynthesis, unsaturated fatty acid biosynthesis, and glycolysis/gluconeogenesis. GO enrichment also highlighted terms related to metabolic processes, catalytic activity, membrane components, and transmembrane transport (Figure 7). These results suggest that AoFlbD affects the metabolic and cellular state associated with nematode-induced development. The connection between metabolism and fungal development has been widely reported in other filamentous fungi. In *Aspergillus* species, secondary metabolism is closely linked to developmental regulation, and the VelB/VeA/LaeA complex coordinates fungal development with secondary metabolite production [42,43]. In the rice blast fungus *M. oryzae*, the mobilization of triacylglycerol and glycogen is required for generating appressorium turgor, showing that metabolic remodeling can directly support infection structure development [44]. In nematophagous fungi, fungal secondary metabolites and nematicidal natural products have also been linked to nematode–fungus interactions [45]. Together, these studies support the idea that metabolic state is closely associated with specialized infection or predation-related developments in fungi. In this study, however, the relationship between extracellular metabolite changes and the increased trap formation or pathogenicity of Δ*AoflbD* remains unclear, because individual metabolites were not functionally tested. Thus, the LC-MS data mainly support metabolic remodeling in Δ*AoflbD*, rather than a direct metabolite-based explanation for enhanced trap formation or pathogenicity. AoFlbB also affected extracellular metabolite profiles, although the changes were weaker than those observed in Δ*AoflbD*.

Overall, AoFlbB and AoFlbD appear to retain the conserved role of Flb-type regulators in promoting sporulation in *A*. *oligospora*, while their deletion led to increased trap formation and pathogenicity. The reduced spore production observed in both mutants indicates that AoFlbB and AoFlbD are required for normal asexual development. In contrast, the enhanced trap formation and pathogenicity suggest that these regulators may also modulate predation-related development under the tested conditions. Phenotypic observations, together with transcriptional and metabolomic analyses, further indicate that AoFlbB and AoFlbD play overlapping but distinct roles. AoFlbB is more closely associated with sporulation, early spore germination, and cell wall organization, whereas AoFlbD has broader effects on pathogenicity, membrane trafficking, extracellular metabolite profiles, and transcriptional responses during nematode induction. Together, these findings broaden the known functions of Flb-type regulators and suggest that Flb-mediated regulation contributes to the coordination of asexual reproduction, cellular remodeling, metabolic remodeling, and trap-mediated predation in NT fungi.

## Data Availability

The data presented in this study are openly available in National Center for Biotechnology Information under the accession number PRJNA1483267.

## Supplementary Materials

The following supporting information can be downloaded at https://www.mdpi.com/article/10.3390/microorganisms14081676/s1: Figure S1: Colony morphology of WT, Δ*AoflbB*, and Δ*AoflbD* strains grown on TG medium supplemented with Congo red or SDS; Figure S2: Hierarchical clustering heatmap of differentially expressed genes (DEGs) in WT and Δ*AoflbD* samples; Figure S3: AoFlbD regulates transcriptional profile during nematode induction in *A. oligospora*. Table S1: Primers used for *AoflbB* and *AoflbD* gene deletion, verification, and RT-qPCR analyses; Table S2. KEGG pathway enrichment analysis of DEGs from Δ*AoflbD* RNA-seq.
