# Supplementary material for: Functional Characterization of AoFlbB and AoFlbD Reveals Their Roles in Sporulation and Trap Formation in Arthrobotrys oligospora
Source: Microorganisms. 2026 Jul 30;14(8):1676. doi: 10.3390/microorganisms14081676 (PMC13515634; doi:10.3390/microorganisms14081676)
Supplement: Supplementary file 1 [file microorganisms-14-01676-s001.zip › microorganisms-4436100-supplementary.pdf]

Supporting Information

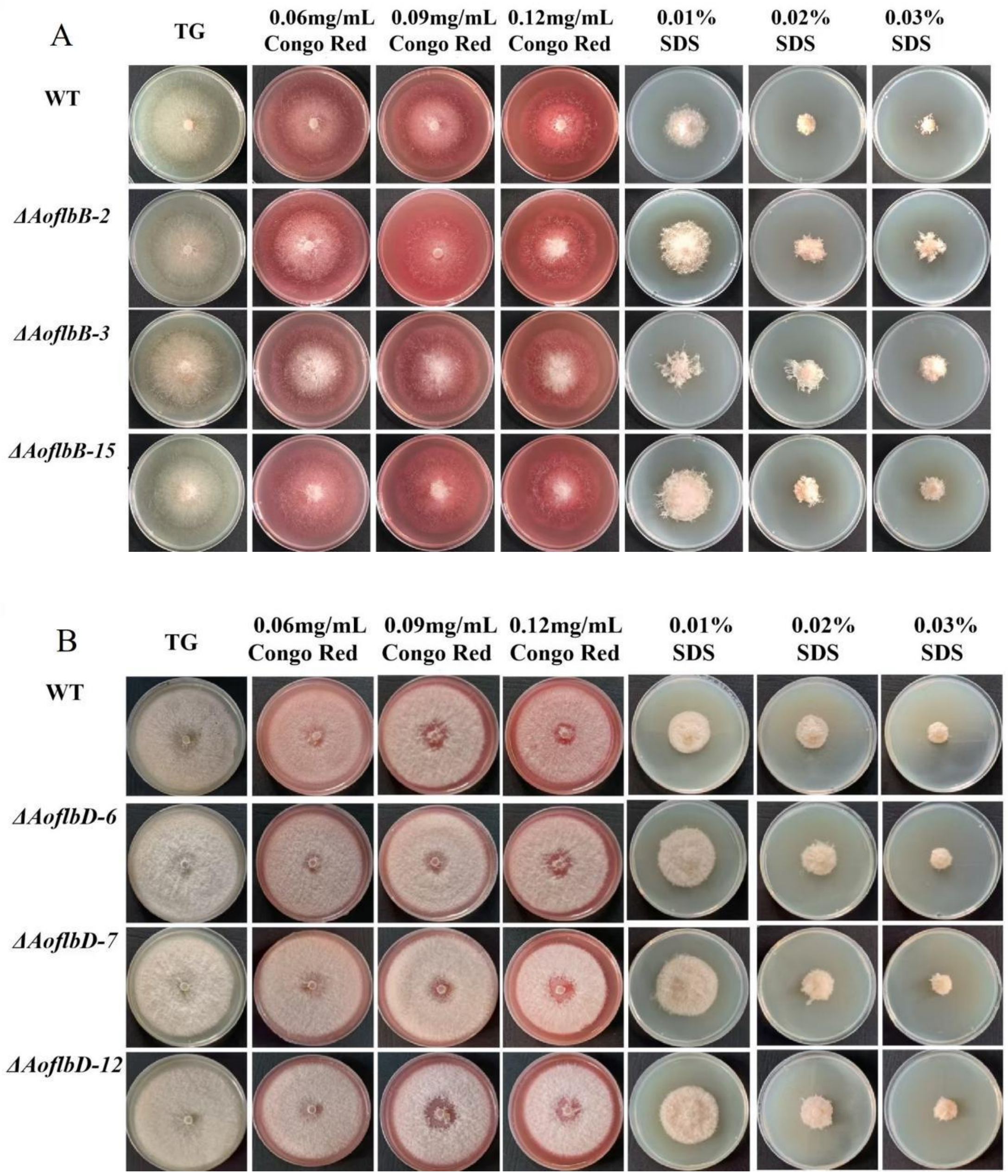

Figure S1. Colony morphology of WT, Δ*AoflbB*, and Δ*AoflbD* strains grown on TG medium supplemented with Congo red or SDS. (A) Colony morphology of WT and Δ*AoflbB* strains. (B) Colony morphology of WT and Δ*AoflbD* strains. The strains were cultured at 28 °C for 5 days.

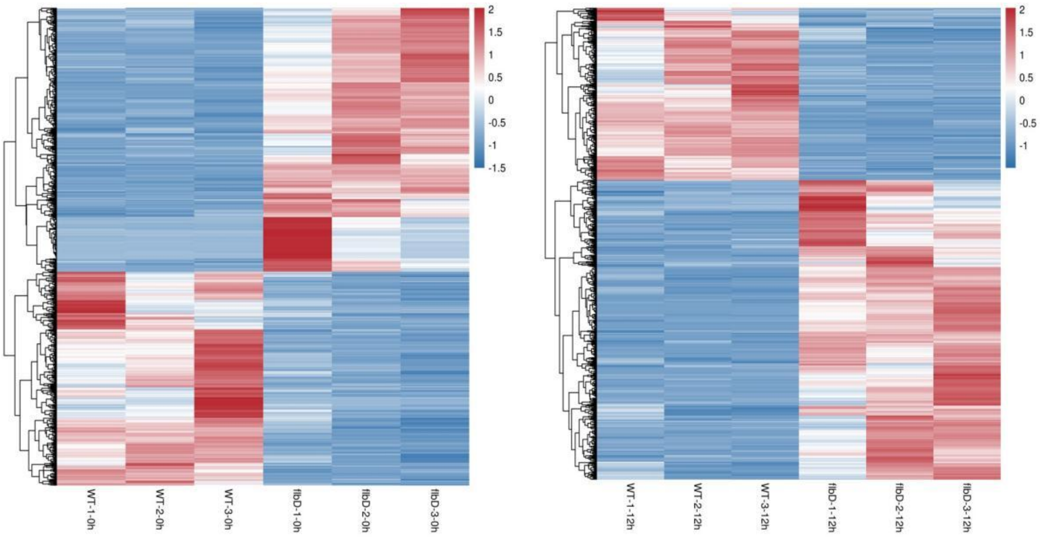

Figure S2. Hierarchical clustering heatmap of differentially expressed genes (DEGs) in WT and Δ*AoflbD* samples under uninduced conditions (0 h) and after 12 h of nematode induction.

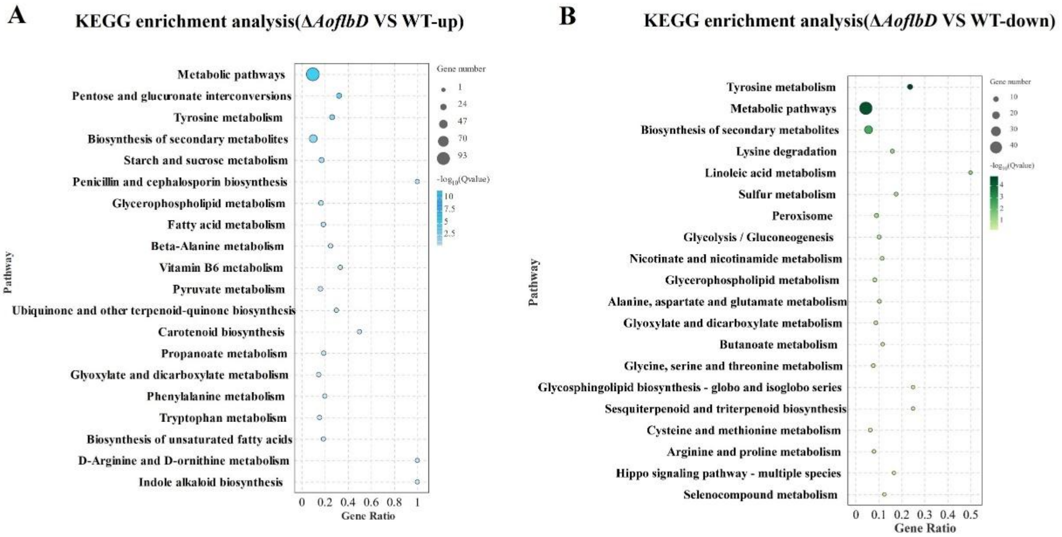

Figure S3. AoFlbD regulates transcriptional profile during nematode induction in *A. oligospora*. **(A, B)** KEGG enrichment analysis of up- (A) and downregulated (B) DEGs in Δ*AoflbD* relative to WT after 12 h of nematode induction.

Table S1. Primers used for *AoflbB* and *AoflbD* gene deletion, verification, and RT-qPCR analyses.

Primer	Sequence (5′-3′)	Purpose/Target
HphF	GTCGGAGACAGAAGATGATATTGAAGGAGC	Amplification of the *hph* cassette
HphR	GTTGGAGATTTCAGTAACGTTAAGTGGAT	
FlbB_ZF	GTAACGCCAGGGTTTTCCCAGTCACGACGGGAGGAGGTGCGGAAGTAA	Amplification of the *AoflbB* 5' homologous arm
FlbB_ZR	ATCCACTTAACGTTACTGAAATCTCCAACTGTGGAGACGAAGGCATAGC	
FlbB_YF	CTCCTTCAATATCATCTTCTGTCTCCGACTTTTCTAGGTGTTTGGGTG	Amplification of the *AoflbB* 3' homologous arm
FlbB_YR	GCGGATAACAATTTCACACAGGAAACAGCTCTTGGGTGGTTGTATCG	
FlbD_ZF	GTAACGCCAGGGTTTTCCCAGTCACGACGGGAAAACTCGGCTGAACCC	Amplification of the *AoflbD* 5' homologous arm
FlbD_ZR	ATCCACTTAACGTTACTGAAATCTCCAACCTCTTCCCTGCCTTTGATTC	
FlbD_YF	CTCCTTCAATATCATCTTCTGTCTCCGACTCTGGGCATTCGGTTTTC	Amplification of the *AoflbD* 3' homologous arm
FlbD_YR	GCGGATAACAATTTCACACAGGAAACAGCTGTAGGTCCAGGCGTAGGAA	
FlbB_YZ-F	GTACTAACGCCGCCTGAA	PCR verification of *AoflbB* deletion
FlbB_YZ-R	AGCCCGCAGAGTGAGAAT	
FlbD_YZ-F	CTCTTACCGCACATCTATC	PCR verification of *AoflbD* deletion
FlbD_YZ-R	AGAAGTTCAGGTCCT	
tubA-F	CCACCTTCGTCGGTAACTC	RT-qPCR internal reference gene, *β-tubulin* (AOL_s00076g640)
tubA-R	TCGTCCATACCCTCACCAG	
317-5F	AATTCCAGCATCGTTAGTGGGT	RT-qPCR of *AoflbB* (AOL_s00078g317)
317-3R	TATCCAACATATGCGACTCCGG	
46-5F	TGACCCAATCACACCAGAGG	RT-qPCR of *AoflbD* (AOL_s00097g46)
46-3R	CGCTCCTGTTCTACCGATGA	
63-5F	AACTTTATGCGCCTTGTCGT	RT-qPCR of *AoabaA* (AOL_s00080g63)
63-3R	TTGGCTAGGTGGTCTGTACG	
120-5F	TCCGGCCCAATGATTCAGAA	RT-qPCR of *AomedA* (AOL_s00210g120)
120-3R	AGATCGCAGGAACATGGTGA	
514-5F	TTGAGGCCTCGATCCGTAGA	RT-qPCR of *AobrlA* (AOL_s00097g514)
514-3R	AGGTAGATGGCGCTGTTACG	
221-5F	TTACATGCCACCCCAAGTCC	RT-qPCR of *AowetA* (AOL_s00173g221)
221-3R	CAATTGCAACTGCGTCCACA	
76-5F	GCCACTCTGCCATCTTTAGC	RT-qPCR of *chs* (AOL_s00078g76)
76-3R	GCATCTTCACCCGCACCAGT	
99-5F	CCATCATCGAGCACACCAAG	RT-qPCR of *gfp* (AOL_s00076g99)
99-3R	CCACCGAGAGTCACTGTCTT	
491-5F	AGCTCTGTTCTGGTGATGCT	RT-qPCR of *gls* (AOL_s00054g491)
491-3R	GATGTTTCGCCAAGGACTCC	
375-5F	GTCATCCTCAAGAACGTCGC	RT-qPCR of *glu* (AOL_s00083g375)
375-3R	ATATGGAAAGTTGGCCGTGC	
89-5F	TACCCTTCTTGACCTCGCTG	RT-qPCR of *hex* (AOL_s00112g89)
89-3R	GAGACGGCGGATGAGTTTTC	
268-5F	CACGTCCATATCACCCTCGA	RT-qPCR of *trs* (AOL_s00097g268)
268-3R	GTGGTATCGGCGACAGTTTC	

Table S2. KEGG pathway enrichment analysis of DEGs from Δ*AoflbD* RNA-seq.

**Comparison:** *flbD*-0h-vs-*flbD*-12h			
**KEGG_A_class**	**Pathway**	**Gene number**	**P value**
Metabolism	Metabolic pathways	49	2.850579e-07
Genetic Information Processing	Nucleocytoplasmic transport	1	0.9439013
Genetic Information Processing	Spliceosome	1	0.9420761
Cellular Processes	Endocytosis	1	0.9004149
Environmental Information Processing	MAPK signaling pathway - yeast	1	0.8869478
Metabolism	Biosynthesis of amino acids	2	0.8687631
Genetic Information Processing	Ribosome biogenesis in eukaryotes	1	0.8675713
Genetic Information Processing	Protein processing in endoplasmic reticulum	2	0.7178193
Metabolism	Glycine, serine and threonine metabolism	1	0.7004118
Metabolism	2-Oxocarboxylic acid metabolism	1	0.6606323
Metabolism	Fatty acid metabolism	1	0.6274275
Metabolism	N-Glycan biosynthesis	1	0.615664
Metabolism	Glycerolipid metabolism	1	0.615664
Metabolism	Valine, leucine and isoleucine degradation	1	0.5910295
Metabolism	Pentose phosphate pathway	1	0.5648423
Metabolism	Arginine and proline metabolism	1	0.537006
Environmental Information Processing	Phosphatidylinositol signaling system	1	0.537006
Metabolism	Steroid biosynthesis	1	0.5224379
Cellular Processes	Peroxisome	2	0.4996933
Metabolism	Methane metabolism	1	0.4919349
Metabolism	Propanoate metabolism	1	0.4759721
Metabolism	Galactose metabolism	1	0.4425512
Metabolism	Fatty acid degradation	1	0.4425512
Metabolism	Folate biosynthesis	1	0.4425512
Metabolism	Pantothenate and CoA biosynthesis	1	0.4250626
Metabolism	Cysteine and methionine metabolism	2	0.4179084
Metabolism	Valine, leucine and isoleucine biosynthesis	1	0.3884495
Metabolism	Biosynthesis of unsaturated fatty acids	1	0.3884495
Metabolism	Histidine metabolism	1	0.3495436

**Comparison:** WT-0h-vs-*flbD*-0h			
**KEGG_A_class**	**Pathway**	**Gene number**	**P value**
Metabolism	Metabolic pathways	91	4.783397e-15
Metabolism	Tyrosine metabolism	15	1.727476e-10
Metabolism	Biosynthesis of secondary metabolites	44	1.149974e-07
Metabolism	Starch and sucrose metabolism	13	1.83433e-05
Metabolism	Glycerophospholipid metabolism	10	0.00014511
Metabolism	Pentose and glucuronate interconversions	8	0.00028627
Metabolism	Pyruvate metabolism	6	0.01196955
Metabolism	Carotenoid biosynthesis	2	0.0157169
Metabolism	Glyoxylate and dicarboxylate metabolism	5	0.03161232
Metabolism	Glycolysis / Gluconeogenesis	5	0.05303572
Metabolism	Butanoate metabolism	3	0.05793819
Metabolism	Alanine, aspartate and glutamate metabolism	4	0.06473941
Metabolism	Glutathione metabolism	4	0.07913471
Metabolism	Sphingolipid metabolism	3	0.08662103
Metabolism	Carbon metabolism	9	0.1022387
Metabolism	Lysine degradation	3	0.144723
Metabolism	Amino sugar and nucleotide sugar metabolism	5	0.1481833
Metabolism	Nicotinate and nicotinamide metabolism	3	0.1575991
Metabolism	Nitrogen metabolism	2	0.1883152
Metabolism	Ascorbate and aldarate metabolism	1	0.1967161
Metabolism	Linoleic acid metabolism	1	0.1967161
Metabolism	Sesquiterpenoid and triterpenoid biosynthesis	1	0.1967161
Metabolism	beta-Alanine metabolism	2	0.2081343
Metabolism	Sulfur metabolism	2	0.2281605
Metabolism	C5-Branched dibasic acid metabolism	1	0.239573
Environmental Information Processing	ABC transporters	2	0.2685318
Metabolism	Biotin metabolism	1	0.2801627
Environmental Information Processing	Hippo signaling pathway - multiple species	1	0.2801627
Cellular Processes	Peroxisome	4	0.3359408

**Comparison:** WT-0h-vs-WT-12h			
**KEGG_A_class**	**Pathway**	**Gene number**	**P value**
Genetic Information Processing	Protein processing in endoplasmic reticulum	1	0.9395197
Environmental Information Processing	MAPK signaling pathway - yeast	1	0.9081681

Genetic Information Processing	Ribosome biogenesis in eukaryotes	1	0.8907931
Metabolism	Metabolic pathways	60	1.526967e-12
Metabolism	Fructose and mannose metabolism	1	0.7140577
Metabolism	Fatty acid metabolism	1	0.6609021
Organismal Systems	Longevity regulating pathway - multiple species	1	0.6722594
Metabolism	Pentose and glucuronate interconversions	1	0.6832417
Metabolism	Alanine, aspartate and glutamate metabolism	1	0.6244509
Metabolism	Pentose phosphate pathway	1	0.5980317
Metabolism	Lysine degradation	1	0.5697822
Metabolism	Steroid biosynthesis	1	0.5549325
Metabolism	Carbon metabolism	11	0.00047763
Metabolism	Terpenoid backbone biosynthesis	1	0.5072851
Metabolism	Fatty acid degradation	1	0.4727621
Metabolism	Glycerophospholipid metabolism	2	0.4749589
Genetic Information Processing	Aminoacyl-tRNA biosynthesis	2	0.4066216
Metabolism	Cyanoamino acid metabolism	1	0.4164613
Metabolism	Histidine metabolism	1	0.3756766
Metabolism	Fatty acid biosynthesis	1	0.285496
Cellular Processes	Peroxisome	3	0.2715455
Metabolism	Glutathione metabolism	2	0.2726341
Metabolism	Glycerolipid metabolism	2	0.2726341
Metabolism	Glycine, serine and threonine metabolism	5	0.0081377
Metabolism	Taurine and hypotaurine metabolism	1	0.235707
Metabolism	Tyrosine metabolism	5	0.00727794
Metabolism	Tryptophan metabolism	2	0.2107545
Metabolism	Nicotinate and nicotinamide metabolism	2	0.2107545
Metabolism	Carotenoid biosynthesis	2	0.0061668

**Comparison:** WT-12h-vs-*flbD*-12h			
**KEGG_A_class**	**Pathway**	**Gene number**	**P value**
Metabolism	Tyrosine metabolism	12	7.553033e-07
Metabolism	Metabolic pathways	92	5.067883e-11
Metabolism	Biosynthesis of secondary metabolites	44	3.183367e-06
Genetic Information Processing	Protein processing in endoplasmic reticulum	1	0.9937744
Metabolism	Oxidative phosphorylation	1	0.9896873
Cellular Processes	Meiosis - yeast	2	0.9301828
Cellular Processes	Phagosome	1	0.8826629
Metabolism	N-Glycan biosynthesis	1	0.8498253
Metabolism	Pentose phosphate pathway	1	0.8078913

Environmental Information Processing	Phosphatidylinositol signaling system	1	0.7827583
Metabolism	Steroid biosynthesis	1	0.7689949
Metabolism	Glycosylphosphatidylinositol (GPI)-anchor biosynthesis	1	0.7388205
Metabolism	Sphingolipid metabolism	1	0.7047403
Metabolism	Biosynthesis of amino acids	6	0.6751625
Metabolism	Fructose and mannose metabolism	2	0.6517274
Metabolism	Sulfur metabolism	1	0.6451833
Metabolism	Cyanoamino acid metabolism	1	0.6227946
Genetic Information Processing	SNARE interactions in vesicular transport	1	0.6227946
Cellular Processes	Mitophagy - yeast	2	0.62115
Metabolism	Histidine metabolism	1	0.5737281
Metabolism	Glutathione metabolism	2	0.5539804
Metabolism	Thiamine metabolism	1	0.4880205
Metabolism	Riboflavin metabolism	1	0.4880205
Metabolism	Various types of N-glycan biosynthesis	2	0.4789381
Genetic Information Processing	Aminoacyl-tRNA biosynthesis	3	0.4543206
Metabolism	Inositol phosphate metabolism	2	0.438673
Environmental Information Processing	MAPK signaling pathway - yeast	5	0.396157
Metabolism	Selenocompound metabolism	1	0.3852452
Cellular Processes	Autophagy - yeast	6	0.3850483
